# The relationship between explicit and implicit attitudes towards drunk driving

**DOI:** 10.1371/journal.pone.0206124

**Published:** 2018-10-22

**Authors:** Laila M. Martinussen, Laura Petranca, Mikael J. Sømhovd

**Affiliations:** 1 Technical University of Denmark, Management Engineering, Kgs. Lyngby, Denmark; 2 Tyrilistiftelsen, Oslo, Norway; Univdersity Hospital of TübingenUniversitatsklinikum Tubingen, GERMANY

## Abstract

Despite downward trends, driving under the influence (DUI) remains one of the most salient traffic safety problems. It is crucial to identify the processes behind a problem behaviour to target the most effective measures to address it. One way of exploring precursors of behaviour is measuring attitudes. All persons hold two types of attitudes, explicit and implicit. Although only one of these (explicit) lays the groundwork for current understandings of DUI, it is imperative to explore both types of attitudes. We explore the relationship between explicit and implicit attitudes towards DUI for the first time in the field. Explicit attitudes (what we say we mean) are measured by a questionnaire. Implicit attitudes (which are introspectively unidentified or inaccurately identified traces of past experience) are measured by the Go/No-go Association Task (GNAT) in a sample of young male drivers (n = 101). The results show a relationship between the two types of attitudes, but not completely in the expected way. Depending whether the amount of alcohol is over or under the legal limit, the relationship between explicit and implicit attitudes varies. We discuss the findings and provide directions for future investigations.

## Introduction

Despite continuous efforts to combat driving under the influence (DUI), it remains one of the most salient traffic safety problems [1–5]. In high-income countries, approximately 20% of fatally injured drivers have excess alcohol in their blood, whereas in low- and middle-income countries, this number may be as high as 69% [4,5]. In Denmark, the yearly cost of DUI accidents is 1.994.800.000 DKK [1,6], making DUI a severe societal and economic problem. Although DUI trends have decreased notably in recent decades [7,8], there is still an urgent need for novel safety initiatives to continue these downward trends.

The literature distinguishes between explicit attitudes and implicit attitudes. Explicit attitudes are the attitudes that we are conscious of and willing to self-report, whereas implicit attitudes are automatized and not immediately consciously accessible. Thus, whereas explicit attitudes are introspectively accessible, implicit attitudes are “introspectively unidentified (or inaccurately identified) traces of past experience” [9]. These traces of experience are associative evaluations resulting from automatic reactions when one encounters a relevant attitude object [9]. Measures of implicit attitudes reveal associative information that people are either unwilling to report or not conscious of and therefore unable to report [10]. Contrary to accessible explicit attitudes measured with self-reports, measures of implicit attitudes require indirect methods that do not rely on introspection, such as computer-based reaction time tasks in which participants associate attitude objects with positive or negative attributes (see [11] for more information). The relationship between explicit attitudes, implicit attitudes and behaviour and the strength of this relationship varies by context. However, explicit attitudes seem to better predict deliberate behaviour, whereas implicit attitudes appear to better predict rapid or spontaneous decision-making [12–14]. A prerequisite for creating efficient DUI interventions is complete understanding of the attitude-behaviour relationship. Therefore, it is necessary to explore all potential processes behind the behaviour. Thus far, no study has explored the role of implicit attitudes towards DUI. Implicit attitudes are essentially memory associations that direct behaviour [9]. For example, when faced with a particular memory cue (e.g., a family member who drinks and drives), thoughts can spontaneously activate the associated behaviour, DUI. The behaviour, DUI, is unplanned and results from the automatic, spontaneous activation of memory patterns [9,15]. Therefore, measuring only explicit attitudes towards DUI overlooks the potential unconscious drivers of the problem.

The fact that drivers explicitly express negative attitudes towards DUI [2] yet DUI accidents are so frequently a factor in traffic accidents indicates a mismatch between attitudes and behaviour, which may be due to the means of measuring and attempting to influence attitudes. Generally, the results of studies of the relationship between explicit attitudes and driving behaviour or behavioural intention are mixed. Some studies have identified a relationship between attitudes and the intention of risky driving behaviour (e.g., [16–23]), whereas other studies have not (e.g., [24,25]). Campaigns and interventions aimed at changing DUI behaviour often attempt to change attitudes as a key element in changing behaviour. Unsurprisingly, as stated by several authors, attitude-changing campaigns have a very limited effect on behavioural outcomes [26,27]. One reason may be insufficient understanding of the attitude-behaviour relationship. Attitude-change efforts primarily aim to change explicit attitudes. Previous studies have suggested that explicit attitudes may not reveal much about DUI because the behaviour is socially stigmatized, thus reducing drivers’ willingness to disclose it [28,29].

Contrary to non-offenders, DUI offenders are more likely to be aware of the potentially negative consequences of DUI [30]. This paradox points to the influence of unconscious cognitions and implicit attitudes in the decision to drive under the influence.

In the context of traffic safety, there is increasing interest in unconscious processes and their effect on behaviour (e.g., [31–36]) because unconscious processes can direct behaviour without conscious awareness [9,37,38]. However, research on implicit attitudes within traffic safety is still in its infancy. There are numerous subfields in the context of traffic behaviour that have yet to be explored, but studies so far show promising results. Sibley and Harré [35] found that traffic safety advertisements affect explicit but not implicit attitudes. The same authors showed that drivers’ self-enhancement bias in relation to driving ability and driver caution was stronger when measured implicitly than explicitly [31], especially among males [36]. Hatfield et al. [32] found negative explicit and implicit attitudes towards speeding. In the same context, Rusu et al. [39] found convergence in implicit and explicit attitudes and suggested that implicit attitudes were able to predict driving violations and traffic accidents. Martinussen et al. [34] found significant relationships between implicit attitudes towards risky and safe driving and self-reported driving skills and aberrant driving behaviour frequency. Furthermore, implicit attitudes predicted observed differences in helmet use and were more robust against social desirability biases than explicit measures [33]. Thus, measuring implicit attitudes towards traffic behaviour is relatively novel in the realm of traffic research but is a promising way of exploring the attitude-behaviour relationship.

Considering the typical effects of alcohol (i.e., impaired ability to consider consequences of actions [40,41]) and that DUI might be spontaneous [19,22], we wanted to explore whether explicit DUI attitudes can predict implicit DUI attitudes. To our knowledge, this is the first study that aims to explore the relation between explicit and implicit attitudes towards DUI. The study will contribute to the literature by increasing understanding of what kind of attitudes (explicit or implicit) drive DUI and may help to inspire new ways to combat the problem.

Our hypothesis is that the inconsistency between DUI attitudes and behaviour is due to a discrepancy between explicit and implicit attitudes. One source of this discrepancy is that respondents tend to answer surveys about DUI in perceived socially acceptable ways [29].

We chose to sample young male drivers because, despite overall road safety improvements and lower accident rates young male drivers, this group still has higher traffic accident rates compared to other groups [42–44]

## Method and materials

### Participants and procedure

We recruited young male students between the ages of 18 to 31 (mean age 22.6) who held a type B driver’s licence for private cars on campus and via a university Facebook page. The participants completed the online Go/No-go Association Task (GNAT) described in 2.1.1 to measure implicit DUI attitudes and an online questionnaire described in 2.1.2 to measure explicit DUI attitudes. The study did not need ethical approval, because according to Danish law, no ethical approval is needed if the study does not use physiological measures, or invasive methods, which this study did not use. Participants were briefed, reminded that they can leave at any time if they want to terminate their participation and they consented orally to participate. The data were analysed anonymously and treated confidentially. Participants were rewarded with a gift card for brunch for participation. Participants were rewarded with a gift card for brunch for participation.

#### The GNAT

The GNAT assesses the strength of association between a target category and two poles of an attribute dimension [11]. The target category represents the studied phenomenon, in this case, pictures of DUI. The evaluative categories are represented by good words (e.g., happy, pleasure) or bad words (e.g., war, catastrophe) on the screen. A second target category, pictures of safe driving, is included as distractor stimuli. It is not important for the tested phenomenon in the present study but is part of the GNAT setup (for detailed information about the procedure, see [11]).

**Fig 1:**
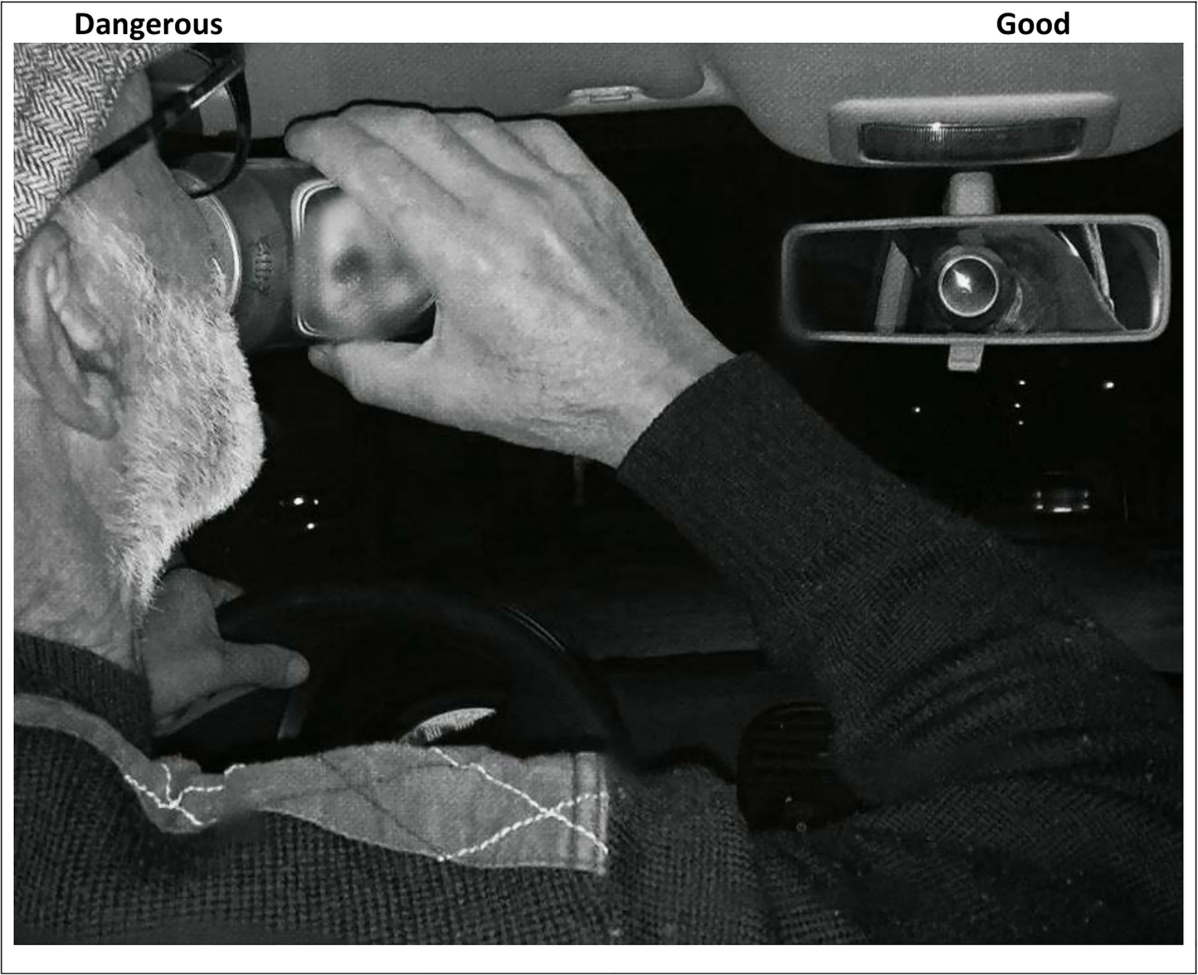
Illustration of the GNAT as seen by the participants. The computer screen presents the target category and attribute dimension on the top right and left. Participants press the spacebar (the “go” response) if the stimulus on the screen belongs to either the target category or the attribute dimension; otherwise, they do nothing (the “no-go” response).

The participants see a target category (*dangerous* representing DUI) or distractor stimuli (*not dangerous* representing safe driving) and an evaluative category (*good* or *bad*) on the right and left side top of the screen (see Fig 1). They are instructed to press the spacebar (the “go” option) if the stimulus on the screen, which may be a picture target or distractor stimuli or an evaluative word stimuli, belongs to either of the categories. If the stimulus belongs to neither of the categories, the participants are to do nothing (the “no-go” option). Fig 1 shows an example of a DUI stimulus that should elicit a “go” response as the left-hand top label is “Dangerous”. The participants is given a short temporal window to respond (750 or 600 milliseconds) before a “no-go” is registered and the program proceeds to the next stimulus. After each trial, the program provides error feedback. Afterwards, the registered “go” and “no-go” responses are converted into scores that reflect whether the response was a hit or a miss. A hit response to the stimulus picture in Fig 1 would be “go” (that is, pressing the spacebar). A no-go, not pressing the spacebar, would be a miss in this instance.

#### Explicit attitude questions

To assess explicit attitudes towards DUI, the participants answered two statements on a ratio scale with the response options “Completely disagree”, “Disagree”, “Neutral”, “Agree”, and “Completely agree”:

1. “I would never drive after drinking alcohol”,
2. “I would never be a passenger in a car where the driver had been drinking alcohol”.

The participants also answered two questions on a six-point Likert scale anchored at “Never” and “Always”:

1. “When it would be preferable and you are under the influence of alcohol but *under* the legal limit, how often would you drive?”
2. “When it would be preferable and you are under the influence of alcohol and are *over* the legal limit, how often would you drive?”

#### GNAT procedure

The GNAT program (Inquisit Millisecond) first runs basic learning of the concept for the program as such, presenting labels and stimuli giving a 1000 millisecond response threshold. Subsequently, four experimental blocks with a 750 millisecond threshold runs in random order prompting response to the four different cues as described above: dangerous, not-dangerous, good, or bad. Finally, four blocks with 600 millisecond threshold is ran in the same way. Order of the cues are randomized. The two main experimental blocks (750 or 600 millisecond) starts with a screen informing the participant that the threshold for response is lowered. Each of the four ‘sub-blocks’ (dangerous, not-dangerous, good, or bad) starts with test 14 trials that is not included in the analyses, which after an information screen are ensued by 60 experimental trials. The experimental blocks comprise combinations of target and evaluative categories as described above.

Analogously, the subsequent computation of implicit attitudes towards DUI is enabled by the difference in sensitivity to the combinations “dangerous/DUI + good” and “dangerous/DUI + bad” blocks. The GNAT presents all blocks twice, which allows the subsequent computation of two independent implicit-attitude scores per participant for the target category (DUI + good; DUI + bad). In line with the GNAT literature, we collected the two independent measures under different response deadlines (750 and 600 ms, respectively). As stated by Nosek and Banaji [11], this procedural variation ensures good reliability and is a particularly conservative strategy (for further discussion, see [11]).

## Analysis

### Scoring procedures

#### GNAT

Following recommendations in the GNAT literature [11], we calculated sensitivity scores from the hit and miss rates (in signal-detection theory, d’) as the performance index; greater scores indicate better performance. From the block performance indices, we then derived implicit attitude scores for the dangerous/DUI target category by computing the difference in performance between associated blocks. For convenience of interpretation, we computed the difference “dangerous/DUI + bad” minus “dangerous/DUI + good” such that greater difference scores consistently represented more socially desirable implicit attitudes towards DUI (i.e., DUI is bad). The distractor blocks (non-DUI stimuli) were not analysed for the rendering of implicit DUI attitudes. The distractor blocks are, however, a conventional part of a ‘balanced’ GNAT procedure, and may additionally be utilized as a baseline condition for the calculation of an alternative implicit attitude coefficient.

#### Explicit measures

We coded the explicit statement data such that higher scores indicated stronger agreement with the statement. For the questions, a higher number indicates that the participant reported that he would perform the behaviour more often.

### Regression model

We entered the explicit variables into an ordinary least squares regression equation with the implicit attitude towards DUI as the independent variable.

## Results

See Table 1. Responding to the statement, “I would never drive after having consumed alcohol”, the median score was four with a mode of five, indicating that most participants claimed that they would never drive when under the influence of alcohol. The statement does not explicitly indicate if this means being under or over the legal alcohol limit (0.5‰). The significant and negative regression coefficient (β = -.08; p = .02) suggests that agreeing to the statement is associated with a less socially desirable implicit attitude towards DUI. The corresponding statement of whether the respondent would be a *passenger* in a car with a driver who had been drinking alcohol is not significant in the model.

**Table 1:** Regression model explicit and implicit attitudes.

	Beta	t	Sig.
When it would be desirable to drive and you have been drinking alcohol but are not over the allowed blood alcohol limit, how often would you drive?	-.236	-2.12	.037
When it would be desirable to drive and you have been drinking alcohol but are over the allowed blood alcohol limit, how often would you drive?	.074	.74	.463
I would never drive after drinking alcohol	-.321	-2.44	.016
I would never drive with someone I knew had drunk alcohol	.213	1.78	.079

When asked specifically how often one would drive when *under* the legal alcohol limit, the mean is 3.7 (SD = 1.8) in an approximately symmetric distribution, showing that many participants would do this often. The significant but negative regression coefficient (β = -.05; p = .04) suggests that being liberal towards driving under the influence, even within the legal limits, is mirrored in a socially undesirable implicit attitude. Answering the question of whether the participant would drive when *over* the legal alcohol limit is not significant in the model. The two questions asking whether the participants would drive when *under* and when *over* the legal limit of alcohol in their blood significantly and positively correlate (see Table 2).

**Table 2:** Correlation between explicit attitude questions and statements.

	**1**	**2**	**3**	**4**
**1**	1	.517**	.439**	.285*
**2**			.234*	-.114
**3**				.302**

Note. Spearmans Correlation, N = 101.

* indicates statistical significance at .05,

** indicates statistical significance at .001.

1 = I would never drive after drinking alcohol

2 = I would never drive with someone I knew had drunk alcohol

3 = When it would be desirable to drive and you have been drinking alcohol but are not over the allowed blood alcohol limit, how often would you drive?

4 = When it would be desirable to drive and you have been drinking alcohol and are over the allowed blood alcohol limit, how often would you drive?

## Discussion

This study explores, to our knowledge, for the first time in the field, the topic of explicit and implicit attitudes towards DUI. The topic is of great importance as the problem with DUI, despite downward trends, remains very salient. New ways to understand and target DUI are therefore crucial. The present results show a relation between explicit and implicit DUI attitudes; however, the results are not convergent.

When asked explicitly, most participants answered that they would never drive after having consumed alcohol. The statement does not clearly indicate if this means being under or over the legal alcohol limit. However, when put into the regression model, the statement was significant and negatively related to implicit DUI attitudes. Thus, agreeing with the statement is associated with a less socially desirable implicit DUI attitude, which initially seems confusing. This puzzling result might have to do with the absoluteness of the statement, which prompts an absolute socially desirable response, “I do not drink and drive”. The implicit attitude measure reveals the “real” attitude or propensity for DUI behaviour. The more “honest” answers actually indicate a more socially desirable implicit attitude. Thus, if the participants answered that they agreed less, their implicit attitude would be more in line with the level of agreement with the statement.

The corresponding statement of whether the respondent would be a *passenger* with a driver who had been drinking alcohol was not significant in the model. The passenger statement was significantly correlated to the statement “I would never drink and drive”, showing that the statements are related.

When asked specifically how often they would drive when *under* the legal alcohol limit, many participants revealed that they often would. This question was negatively significant in the model. Thus, the more often the participants said they would drive when under the legal limit, the more positive implicit DUI attitudes they held. Thus, the participants were liberal towards DUI (when under the legal limit), which mirrors a less social desirable implicit attitude. Accordingly, even if it is legal and socially acceptable, the tendency to be willing to drink and drive when an individual believes he or she is under the legal limit is in itself a socially undesirable implicit and explicit attitude. As drivers’ poorly judge safe drinking levels (the amount of alcohol intake to be under the legal limit) [45], their liberal attitudes towards driving when under the legal limit are of concern.

Answering the question of whether the participants would drive when *over* the legal alcohol limit was not significant in the model. The questions about being over and under the legal limit were, however, significantly and positively correlated, further suggesting a link between willingness to drive when under the legal alcohol limit and unfortunate explicit DUI attitudes. The question about driving over the legal limit resembles the statement “I would never drink and drive” in its absoluteness. Most drivers are aware that drunk driving (over the legal limit) is prohibited, leading participants to answer in socially acceptable ways (e.g., [29]). However, when we test their implicit attitudes, it seems they are not completely against DUI as they would drive when they perceive themselves to be under the legal limit. A complete and absolute negative DUI attitude is thus not observed in the present sample.

These findings replicate and extend earlier findings regarding alcohol associations [46,47]: people hold both negative and positive associations with alcohol. In addition, Wiers et al. [46,47] show that participants hold both arousal and sedation associations with alcohol. In relation to our study, depending on the amount of alcohol consumed, participants hold both positive and negative implicit and explicit DUI attitudes. Similar findings are observed in Baum [45]: people who drive under the influence have more accepting DUI attitudes than the general community when asked about driving under the legal limit, but they are against drunk driving when asked promptly and absolutely.

## Limitations, summary and conclusion

A limitation of this study is the generalizability; the study explored only young male drivers, which was a deliberate choice as this group is the most accident-prone. However, the sample was Danish and mainly male students where some of them were Facebook users and was recruited from there. Thus, the generalizability is restricted to young male Danish students. Future studies should explore other groups of drivers, preferably those that have already driven under the influence.

Implicit attitudes are automatic and mostly outside of conscious control [9]. They may therefore be more “truly” predictive of actual behaviour when considering socially sensitive issues [48,49]. Although the participants hold negative explicit attitudes towards DUI (when over the legal limit), their implicit attitudes may nonetheless reveal a tendency towards DUI behaviour. When asked about the probability of driving when under the legal limit, the liberal attitude towards this behaviour is mirrored in the implicit measure. The correlation between the questions about driving below and above the legal limit suggests a liberal attitude towards DUI regardless of whether the driver is within the legal limit. Thus, these attitudes may reflect a rise in the propensity for actual DUI behaviour. When prompted for an absolute answer to the propensity for DUI, respondents answer in a socially desirable way, understanding the question as meaning “real” DUI (i.e., over the legal limit). As this study addresses the relationship between explicit and implicit DUI attitudes for the first time, more research is needed to compare the attitudes and results of different groups of drivers. However, this study is a promising first take on the issue and lays the groundwork for further investigation. Future studies should explore the relation between explicit and implicit DUI attitudes in other groups of drivers and in other driving cultures. As the effect of implicit processes has been studied within other areas of psychology for decades, the traffic psychology community has a large amount of results upon which to rely. However, few studies have targeted traffic problems by considering both explicit and implicit attitudes.

## Supporting information

S1 Dataset (SAV)
